# The Different Classification of Hospitals Impact on Medical Outcomes of Patients in China

**DOI:** 10.3389/fpubh.2022.855323

**Published:** 2022-07-18

**Authors:** Lele Li, Tiantian Du, Siyu Zeng

**Affiliations:** ^1^School of Labor and Human Resources, Renmin University of China, Beijing, China; ^2^Research Office of Medical and Care Insurance, Chinese Academy of Labour and Social Security, Beijing, China; ^3^School of Logistics, Chengdu University of Information Technology, Chengdu, China

**Keywords:** classification of hospital, medical outcomes, nested multinomial logit model, disease types, a tiered delivery system

## Abstract

**Background:**

In China, different classification of hospitals (COH) provide treatment for patients with different degrees of illness. COH play an important role in Chinese medical outcomes, but there is a lack of quantitative description of how much impact the results have. The objective of this study is to examine the correlation between COH on medical outcomes with the hope of providing insights into appropriate care and resource allocation.

**Methods:**

From the perspective of the COH framework, using the Urban Employee Basic Medical Insurance (UEBMI) data of Chengdu City from 2011 to 2015, with a sample size of 512,658 hospitalized patients, this study used the nested multinomial logit model (NMNL) to estimate the impact of COH on the medical outcomes.

**Results:**

The patients were mainly elderly, with an average age of 66.28 years old. The average length of stay was 9.61 days. The female and male gender were split evenly. A high level of hospitals is positively and significantly associated with the death and transfer rates (*p* < 0.001), which may be related to more severe illness among patients in high COH.

**Conclusion:**

The COH made a difference in the medical outcomes significantly. COH should be reasonably selected according to disease types to achieve the optimal medical outcome. So, China should promote the construction of a tiered delivery system.

## Background

**Figure 1:**
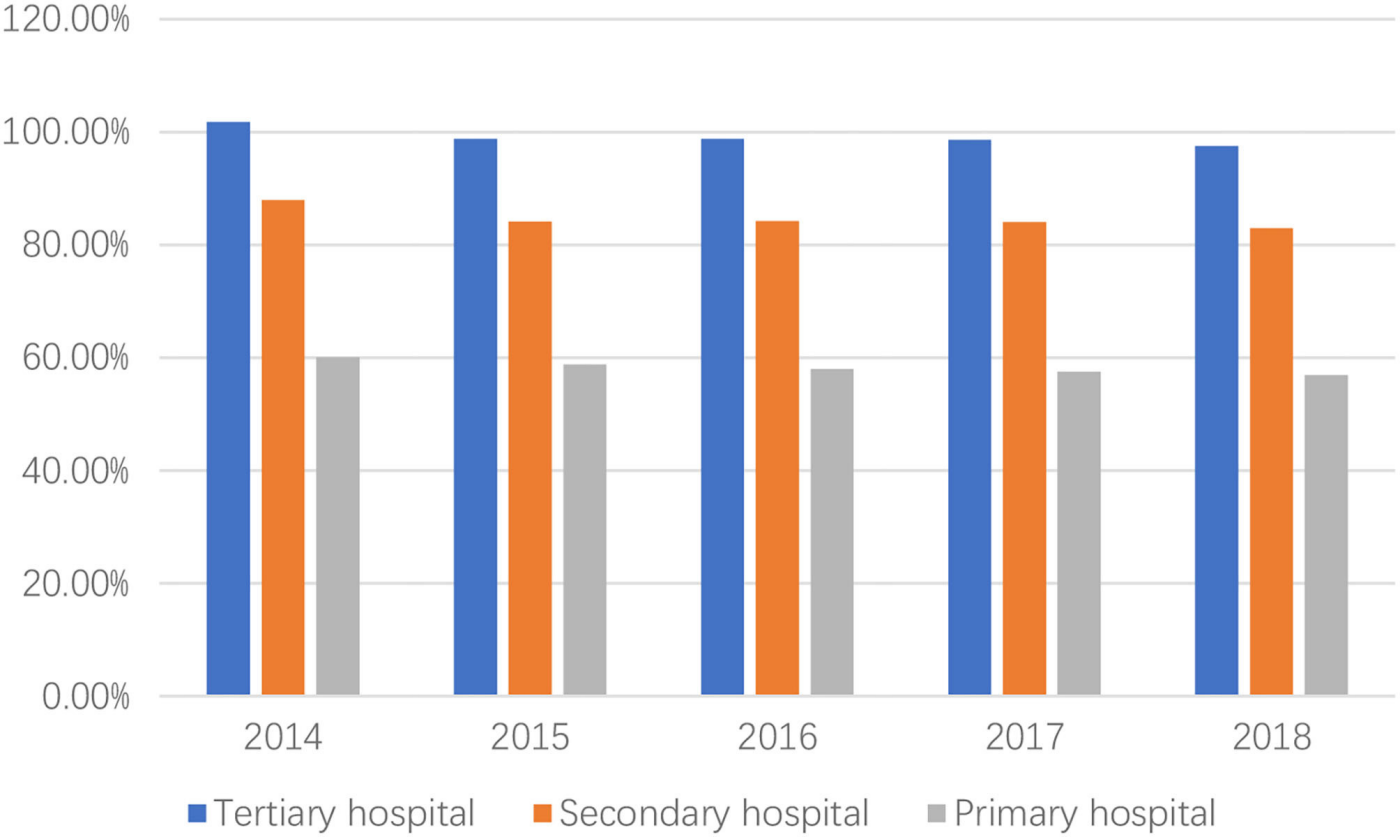
The utilization rate of bed in China. Data source: China Health Statistical Yearbook: 2014–2018.

Improving the quality of medical services has become an important goal and critical issue in healthcare reform worldwide. In China, the government announced the implementation of a Healthy China strategy, which placed people's health improvement as the primary strategic goal of the health system. But for a long time, medical resources were in short supply in China. Furthermore, the phenomenon that hospitals have inverted pyramid structural characteristics had existed for a long time in China (1). According to Table 1, the total number of outpatients and emergency patients in tertiary hospitals keeps increasing, from 1.4 billion in 2014 to 1.85 billion in 2018, with an increase of 32% over 4 years. While the number of patients in primary healthcare institutions has been stable at about 4.4 billion. The mismatch of medical resources and the imbalance of supply and demand make the problem of inaccessible medical care. Lining up for 3 h to see a doctor for 3 min and overburdened doctors are the characteristics of tertiary hospitals (2). According to Figure 1, the utilization rate of beds in tertiary hospitals has been overloaded, reaching 97.5% in 2018, while that of primary hospitals is only 56.9%.

**Table 1:** Hospital outpatient visits: China, 2014–2018.

**Hospital types**	**Outpatient visits (x10** ^**8**^ **)**				
	**2014**	**2015**	**2016**	**2017**	**2018**
Public hospital	26.5	27.1	28.5	29.5	30.5
Private hospital	3.2	3.7	4.2	4.9	5.3
Tertiary hospital	14.0	15.0	16.3	17.3	18.5
Secondary hospital	11.5	11.7	12.2	12.7	12.8
Primary hospital	1.8	2.1	2.2	2.2	2.2
Primary health care institutions	43.6	43.4	43.7	44.3	44.1

*Data source: China Health Statistics Yearbook: 2014–2018*.

Therefore, the Chinese government promotes the construction of a tiered delivery system. By establishing a tiered delivery system, the diagnosis and treatment system of slight illness in the community, serious diseases in the hospital, and rehabilitation back to the community can be divided to meet the health needs of people with different types of diseases. However, the sense of better medical outcomes in big hospitals has always been rooted in the hearts of the people, which is not conducive to the promotion of a tiered delivery system. Therefore, it is an important research subject for the Healthy China strategy to analyze the impact of classification of hospital (COH) on medical outcomes and to put forth suggested solutions to promote the construction of a tiered delivery system.

In 1989, the former Ministry of Health enacted the measures for the administration of the hospital grade (trial draft) which implemented COH in China to provide clarity and structure. The COH divided hospitals into Grade I, II, and III hospitals according to their functions and roles. Grade I hospitals include community health centers and township health centers that directly provide prevention, medical care, and rehabilitation services to residents. Grade II hospitals are secondary hospitals that provide comprehensive medical services to a region and undertake some teaching, and scientific research tasks. Grade III hospitals are tertiary hospitals that provide high-level specialized medical services and undertake advanced teaching and scientific research tasks. Further, these three grades are subdivided into three subsidiary grades, including A, B, and C grades based on the hospital's scale, service provision, medical technology and equipment, medical research, and so on.

Classification of hospital has played an important role in establishing an efficient healthcare administration system, and in strengthening the three-tier prevention healthcare network, providing convenient and suitable medical services. Therefore, it is the intention of this analysis to examine the effect of COH on medical outcomes; as there have been no studies exploring this effect; especially in China. For the purposes of this study, hospitals were divided into three levels, such as Grade II Level A hospitals, Grade II Level B hospitals, and Grade II Level C hospitals. In this study, hospitals with a grade II basic classification were selected as the research objects. Grade II hospitals have the largest number and are most representative in China. Grade II hospitals (101–500 beds) are regional hospitals that provide comprehensive medical and health services to multiple communities and undertake certain teaching and research tasks. Grade II hospitals include three grades: Grade II Level A, Grade II Level B, and Grade II Level C. Specific grading criteria and patient selection are shown in the Table 2.

**Table 2:** The characteristics of different classifications of hospitals (COH).

**COH**	**Definition**	**Evaluation score**	**Patient characteristics**
Grade II Level A	Determined by grade evaluation according to the comprehensive level of hospital functions, tasks, facilities, technical construction, medical service quality and scientific management. It is the strongest hospital in grade two hospitals.	The grading standard assessment must reach more than 900 points	Generally severe patients
Grade II Level B	Basically point to hospital of average city, county provincial city area level hospital, and the worker hospital of unit of industrial mine of comparable scale, enterprise or institution	The standard assessment should range from 750 to 899 points	Patients with common diseases
Grade II Level C	General city, county hospitals and provincial city level hospitals, as well as a considerable scale of industrial and mining enterprises and institutions of the staff hospitals	The grading standard assessment is below 749 points	Mild disease

## Literature Review

The effect of COH on medical outcome has a significant difference in terms of income, and different income groups are influenced by different levels of hospitals. A large number of scholars have done relevant studies on Chinese COH. Li (3) believed that the limited medical resources of tertiary hospitals are seriously occupied by common diseases and chronic diseases, and more than 60% of patients in tertiary hospitals can be treated in grassroots medical institutions. Li et al. (4) studied the data from the New Rural Cooperative Healthcare system and found that high-income groups increased the utilization of medical services more significantly. At the same time, income difference have a significant impact on health status. Liu and Hu (5) concluded that the level of health inequality in China is more beneficial to high-income people.

There are many factors influencing therapeutic outcomes, such as hospital size, hospital types, hospital service capacity, hospital accreditation, hospital competition, and medical insurance. Researchers also care about the effect of COH on medical outcome in the price and distance. Medical outcome refers to the impact of medical services or medical treatment on patient's life. The classical Donabedian framework for evaluating remedial services is divided into structural, process, and outcome indicators (6). Another classical approach is the five D's, which include death, disease, disability, discomfort, and dissatisfaction (7). However, the two classical model was used less in contemporary outcome research. There are three kinds of measures for medical outcomes in recent research, which are the economic outcome model (8), the clinical outcome model (9, 10), and the humanistic outcome model (11, 12). The higher the level of hospitals, the higher the price of healthcare services. Qian et al. (13) studied the medicinal demand behavior of rural residents and found that the price elasticity of low-income groups was higher than that of high-income groups. Han (14) held an opinion that when patients pay attention to medical service quality or suffer from serious diseases. They will consider higher-level hospitals for treatment. The research on the relationship between hospital volume and medical outcome has just begun in China. Taking colorectal cancer surgery as an example, Ma (15) systematically reviewed the clinical data of more than 1 million cases of colorectal cancer patients reported by 10 centers from 1999 to 2011 and discussed the effect of the hospitals' or surgeon's volume on the complications and prognosis of patients with colorectal cancer. The results showed that the volume of operation did play a role in the restorative outcome, and the high volume of operation of hospitals and surgeons can reduce the rate of complications and tumor recurrence and better improve the prognosis of patients.

Some scholars have estimated the effect of hospital size on medical outcomes, with mixed consequences. Sjetne et al. (16) used the Patients' Experience Questionnaire (PEQ) to study the effect of hospital size and type on hospital care in 50 Norwegian hospitals. They found hospital size and type have an influence on patient experience and medical outcome. Reinikainen et al. concluded the same results (17).

There is a major empirical literature on the relationship between hospital types and medical outcome. There are two dominant type categories. First, they separated the hospital into teaching hospitals and non-teaching hospitals. Most researchers found that teaching status does affect the medical outcomes and teaching hospitals offer better medical outcomes (18–23). In the contrary, Fleming et al. (24) found lower mortality rates in non-teaching hospitals than in teaching hospitals. Some researchers also concluded there are no difference of medical outcomes between teaching and non-teaching hospitals (25–28). Second, they classified hospitals into government-owned hospitals, for-profit hospitals, and not-for-profit hospitals according to their ownership. A systematic review of the literature reports mixed results: whether hospitals ownership impacts the medical outcome, as measured by mortality rates and other adverse events, depends on the region, the data source, and the period of analysis (29). Many studies have found that hospitals ownership does matter the medical outcomes. According to some studies, private hospitals have better therapeutic outcomes (30–33). In the meanwhile, some studies found that not-for-profit hospitals have better therapeutic outcomes (34–36) and some found that for-profit hospitals have better medical outcomes (37). However, in America, some researchers also find no difference in therapeutic outcome by ownership type (38–41).

Studies on the relationship between hospital service capacity and medical outcomes have been conducted for more than 30 years, but there is still no definite conclusion. Many scholars take some acute diseases or operations as examples to study the correlation between them. The research results are mainly divided into two categories: one is that the hospital service volume is positively related to the curative outcome (42) and the other is that the two are irrelevant (43, 44). Meanwhile, there are also some studies on the correlation between hospital accreditation and medical outcome. Most of the research results show that hospital accreditation can improve healing outcomes (45, 46).

Numerous scholars have examined the relationship between hospital competition and medical outcome. The evidence is mixed. Kessler and McClellan (47) and Hugh et al. (48) found a positive effect of competition on medical outcomes. However, Shortell and Hughes (49) and Mukamel et al. (50) found no effect. Propper et al. (51) exploited a policy change by the UK government in the 1990's and found that competition was associated with worse medical outcomes. Gowrisankaran and Town (52) concluded the same results as Propper et al. In addition, the study about medical insurance has been a long time coming. Among them, most of the results showed that the medical insurance does impact medical outcomes (53–55).

In summary, there is no relevant research on the effect of COH on medical outcome. Most scholars focus on hospital scale, hospital types, hospital volume, hospital accreditation, and medical insurance. While Chinese scholars focus more on healthcare service utilization, healthcare demand, and healthcare seeking behavior. The possible reasons are: (a) medical outcome is a very difficult index to measure, so far there is no clear measurement of medical outcome. (b) The majority of the healthcare systems lack a strict COH. The hospital is different in terms of scale and ownership type. Furthermore, such as the United Kingdom and the United States, the range of hospitals is a high degree of homogenization because of advanced healthcare systems. At the same time, the family doctor system makes patients unable to choose doctors and hospitals freely, so the effect of COH is relatively weak. However, under COH, as a Chinese characteristic, hospitals are divided into different levels in a more detailed way, and there is a large gap in the quality and capability of healthcare service between different levels, which has a significant impact on the medical outcome. (c) There are many factors affecting the therapeutic outcome, which cannot be included, and relevant data are difficult to obtain. Therefore, this article is just an exploratory study on the effect of COH on medical outcomes.

## Methods

### Data Source and Variable Descriptions

The data were derived from UEBMI s of Chengdu City from 2011 to 2015. The sample size was 1,035,556 hospitalized patients. Due to the large number of missing values in the transition state, the sample size is reduced to 512,658 after removing the missing values. We used the software of Stata to analyze the data.

The dependent variable used was medical outcome, which consisted of death, transfer, rehabilitation, and no cure. Medical outcomes were measured as changes in a patient's health and can be used to generate evidence about the medical benefits of medical services (14). The death referred to patients who were not cured in medical institutions and lose their lives; transfer referred to the patients treated from one hospital to another hospitals; and rehabilitation meant that patients who were cured in medical institutions and recovery function; others were not cured, which referred to the other conditions besides death, transfer, and rehabilitation. Therefore, rehabilitation was invoked as the reference group for the model. The independent variables were the factors that affected patients' therapeutic outcomes, such as hospital characteristic factors, patient characteristic factors, and potential influencing factors. Individual characteristic factors included age, gender, and length of stay. After communication with doctors, we classified all the diseases in the article into three categories according to the International Diagnostic Code (ICD)-10: chronic diseases, critical diseases, and common diseases, and chronic diseases were considered as the reference group.

### Model Structure

Referring to existing literature, this article sets the model as follows:


$$\begin{array}{l} \text{prob}\left(\text{resul}{\text{t}}_{\text{i}}\right)={\text{β}}_{0}+{\text{β}}_{1}\text{ran}{\text{k}}_{\text{i}}+{\text{β}}_{2}{\text{X}}_{\text{i}}+\varepsilon_{\text{i}} \end{array}$$

Where, *i* represents the *i* patient in the sample, *result*_*i*_ represents the medical outcome of the *i* patient, *rank*_*i*_ represents the effect degree of the COH on the *i* patient, *X*_*i*_ represents a group of observable control variables, and ε_*i*_ represents the error term.

The multinomial logit model (MNL) is a classic model for healthcare demand behavior (1), healthcare choice (2), and other issues. The dependent variables of the nested MNL (NMNL) model mainly have two characteristics, which are different from the general MNL model. First, the dependent variables change with individual changes, such as gender, age, and consumption. Second, the dependent variables change with the overall changes, such as programs, policies, and projects. There are many factors affecting the medical outcome of patients. On the one hand, the curative effect of patients is affected by individual characteristics, such as gender and age. On the other hand, they are affected by the medical institution's characteristics, such as the price, medical equipment, medical level, and social reputation, which reflects the heterogeneity of medical institutions. The general MLN model can only deal with the first type of variables that change with characteristic changes. They cannot deal with the second type of variables that change with the overall changes. However, NMNL model can meet the need of dealing with two types of variables at the same time, which is more suitable to analyze the influencing factors of medical outcome.

## Results

### Descriptive Statistics

The COH is an order variable in China, and to better observe the characteristics of COH, we directly coded it as categorical variable for statistical description in Table 3. As shown in Table 3, rehabilitation accounted for the highest proportion in the treatment outcome, and that grade II Level A hospitals accounted for the highest proportion of hospitals. The patients were mainly elderly, with an average age of 66.28 years old. The average length of stay in a hospital was 9.61 days. The female and male gender were split evenly. In terms of reimbursement ratio, the actual reimbursement ratio of Grade II Level A hospitals was higher than that of Grade II Level B hospitals and below. Surprisingly, the reimbursement ratio of Grade II Level B was the lowest among all hospitals. In terms of medical expenses, the average medical expenses of Grade II Level A hospital were the highest. The medical institutions with the lowest average medical expenses were the Grade II Level B hospital. In addition, the average medical expenses of the Grade II Level C hospital were in the middle, which may be related to more prescriptions from grassroots institutions.

**Table 3:** Variable definition and descriptive statistics.

**Variable type**		**Variable name**	**Variable definition**	**Mean**	**SD**
**The dependent variable**					
Medical effect	Treatment outcome	Death	Death = 1, otherwise = 0	0.01	0.11
		Transfer	Transfer = 1, otherwise = 0	0.03	0.12
		Rehabilitation	Rehabilitation = 1, otherwise = 0	0.91	0.29
		No cure	No cure = 1, otherwise = 0	0.05	0.21
**The independent variables**					
Hospitals	COH	Grade II Level A	Grade II Level A = 1, otherwise = 0	0.83	0.37
		Grade II Level B	Grade II Level B = 1, otherwise = 0	0.12	0.33
		Grade II Level C	Grade I = 1, otherwise = 0	0.03	0.16
**The control variables**					
Potential influencing factors	Reimbursement ratio	Grade II Level A	Average reimbursement ratio in Grade II Level A hospital	0.71	0.16
		Grade II Level B	Average reimbursement ratio in Grade II Level B hospital	0.64	0.15
		Grade II Level C	Average reimbursement ratio in Grade I hospitals	0.69	0.17
	Medical expenses	Grade II Level A	Average medical expenses in Grade II Level A hospital	6,024.85	6,527.37
		Grade II Level B	Average medical expenses in Grade II Level B hospital	3,917.81	4,000.12
		Grade II Level C	Average medical expenses in Grade I hospital	4,453.76	3,849.22
Individual characteristic factors		Gender	Female = 1, others = 0	0.52	0.50
		Age	Age of participant	66.28	23.81
		Length of stay in hospital	Actual length of stay	9.61	43.42
Disease types		Chronic disease	Chronic disease = 1, otherwise = 0	0.05	0.21
		Critical disease	Critical disease = 1, otherwise = 0	0.06	0.23
		Common disease	Common disease = 1, otherwise = 0	0.89	0.30

### Empirical Results

**Figure 2:**
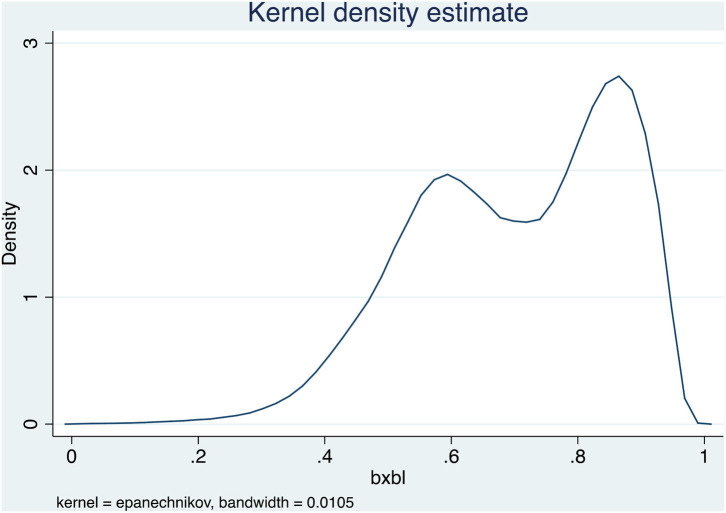
The density chart of reimbursement rates of medical insurance for different hospitals grades.

To better facilitate interpretation and meaningful analysis, we refer to the treatment mode of the Likert scale and treat the sequential variable COH as continuous variable in the regression model. When COH is a continuous variable, Grade II Level C, Grade II Level B, and Grade II Level A are assigned as 1, 2, and 3, respectively. According to the empirical results (Table 4), the COH has a significant correlation with the therapeutic outcome, but the influence degree and direction of different outcome states were not consistent. COH was significantly positively associated with death and transfer (*p* < 0.001), but that no cure was significantly negative (*p* < 0.001), which may be related to more severe illness among patients in high-level hospitals. Reimbursement rates were mainly between 60 and 95%, and there were sizable differences in reimbursement rates among different hospitals grades (Figure 2). However, no more detailed reimbursement rates were set for different disease types. The effect of medical expenses on the treatment outcome was significantly positive (*p* < 0.001), which indicates that medical expenditures had a significant impact on the medical outcome. The more medical expenses, the better the therapeutic outcome was likely to be. In addition, distinct characteristics had a significant correlation with therapeutic outcomes. Gender was significantly negatively associated with death and transfer, and significantly positively associated with no cure, indicating that gender had an important correlation with medical outcomes. In terms of death and transfer, female had a better medical outcome than male. The correlation with age on death and transfer is markedly positive, while the correlation with no cure was negative, indicating that the older the age, the higher the probability of death and transfer. The length of stay in the hospital had a negative correlation with medical outcome, indicating that the more days in the hospital was not necessarily correlated with the better medical outcome. The disease type on death and no cure was significantly negatively associated with medical outcome, indicating that the more severe the disease type was, the higher the probability of death and no cure. Hospital transfers may occur spontaneously or involuntarily.

**Table 4:** The nested multinomial logit (NMNL) model empirical results.

**Variables**	**Treatment outcome**			
	**Medical effect**	**Death**	**Transfer**	**No cure**
COH	0.3271^***^ (10.35)	0.4817^***^ (14.65)	1.8231^***^ (101.15)	−0.3392^***^ (−13.14)
Reimbursement ratio		−1.0619^***^ (-11.58)	−1.2951^***^ (−18.50)	6.4000^***^ (87.48)
Medical expenses		0.6125^***^ (30.36)	0.0745^***^ (4.49)	0.1513^***^ (10.25)
Gender		−0.4608^***^ (−18.16)	−0.0760^***^ (−4.42)	0.0691^***^ (5.16)
Age		0.0388^***^ (46.06)	0.0088^***^ (21.76)	−0.0018^***^ (−4.52)
Length of stay in hospital		−0.0002 (−0.20)	−0.1284^***^ (−41.14)	−0.0036^***^ (−3.68)
Disease type		−0.1268^***^ (−5.46)	0.0125 (0.60)	−0.2248^***^ (−20.43)
Constant term	−4.6342^***^ (−117.17)	−12.5374^***^ (−64.27)	−5.3939^***^ (−42.47)	−8.2196^***^ (−66.98)
Log likelihood	−193,657.9	−179,366.71		
Prob>chi^2^	0.0000	0.0000		
Pseudo *R*^2^	0.0343	0.1056		

*** *Significant at 1%*.

After distinguishing the COH (classification of hospital), according to the empirical results (Table 5), the Grade II Level A hospitals on death and transfer were significantly negatively associated with outcomes (*p* < 0.001), while those on no cure were significantly positive (*p* < 0.001). The Grade II Level B hospitals on death and no cure were significantly negatively associated with outcomes (*p* < 0.001), and the Grade II Level B hospitals and transfer were significantly positive (*p* < 0.001). The Grade II Level C hospitals on death and transfer were significantly positively associated with outcomes (*p* < 0.001), while the correlation with no cure was significantly negative (*p* < 0.001). It indicates that COH had an important association with the medical outcome. From a horizontal perspective, hospitals of the same level have different associations with different medical outcomes. This may be related to the type and severity of the disease. Different levels of hospitals have different associations with medical outcomes. It is not that the higher the level of hospitals, the better the medical effect.

**Table 5:** The NMNL model empirical results [by classification of hospital (COH)].

**Variables**	**Treatment outcome**								
	**Death**	**Transfer**	**No cure**	**Death**	**Transfer**	**No cure**	**Death**	**Transfer**	**No cure**
COH									
Grade II Level A hospitals	−0.42652^***^ (−13.06)	−1.8738^***^ (−106.61)	0.4087^***^ (15.81)						
Grade II Level B hospitals				−0.8093^***^ (−14.04)	1.2156^***^ (68.99)	−0.3141^***^ (−10.86)			
Grade II Level C hospitals							1.7973^***^ (46.75)	1.8962^***^ (68.47)	−0.2735^***^ (−5.25)
Reimbursement ratio	−1.1098^***^ (−12.15)	−1.1074^***^ (−16.46)	6.3768^***^ (87.27)	−1.3412^***^ (−14.84)	−1.1129^***^ (−16.74)	6.4322^***^ (87.76)	−1.3619^***^ (−14.80)	−1.2963^***^ (−19.74)	6.5130^***^ (89.33)
Medical expenses	0.5612^***^ (31.69)	0.0298* (1.87)	0.1621^***^ (11.03)	0.4904^***^ (27.38)	−0.2574^***^ (−16.19)	0.1901^***^ (13.02)	0.5974^***^ (34.73)	−0.2778^***^ (−17.63)	0.1939^***^ (13.31)
Gender	−0.4553^***^ (−17.97)	−0.0729^***^ (−4.40)	0.0698^***^ (5.21)	−0.4534^***^ (−17.89)	−0.0456^***^ (−2.78)	0.0696^***^ (5.20)	−0.4804^***^ (−18.86)	−0.0445^***^ (−2.71)	0.0708^***^ (5.29)
Age	0.0384^***^ (45.97)	0.0082^***^ (21.32)	−0.0017^***^ (−4.44)	0.0394^***^ (47.70)	0.0105^***^ (27.86)	−0.0022^***^ (−5.64)	0.0355^***^ (42.88)	0.0111^***^ (30.19)	−0.0018^***^ (−4.52)
Length of stay in hospital	0.0056^***^ (9.12)	−0.1241^***^ (−40.88)	−0.0036^***^ (−3.64)	0.0065^***^ (9.34)	−0.0942^***^ (−31.99)	−0.0054^***^ (−5.33)	0.0044^***^ (9.59)	−0.1022^***^ (−36.50)	−0.0047^***^ (−4.67)
Disease type	−0.1270^***^ (−5.48)	0.0172 (0.85)	−0.2243^***^ (−20.40)	−0.1234^***^ (−5.33)	0.0209 (1.05)	−0.2264^***^ (−20.58)	−0.1394^***^ (−5.94)	0.0185 (0.93)	−0.2276^***^ (−20.72)
Constant term	−11.1833^***^ (−68.36)	−1.4506^***^ (−13.14)	−9.0475^***^ (−80.10)	−10.8116^***^ (−64.67)	−1.0686^***^ (−9.52)	−8.8721^***^ (−77.57)	−11.4566^***^ (−70.96)	−0.6036^***^ (−5.43)	−8.9898^***^ (−79.39)
Log likelihood	−182,970.08			−186,460.96			−186,296.56		
Prob>chi^2^	0.0000			0.0000			0.0000		
Pseudo *R*^2^	0.1095			0.0925			0.0933		

*** *Significant at 1%*.

After distinguishing disease type, according to the empirical results (Table 6), the association between COH and medical outcome was significant, the association with COH between death and transfer was significantly positive (*p* < 0.001), and the association with no cure was significantly negative (*p* < 0.001). The type of disease influenced the medical outcomes. Taking chronic disease as the reference group, the effect of critical disease on the medical outcome was positive, indicating that the higher the severity of the disease, the higher the probability of death and transfer. Disease types influenced medical outcomes. Distinct types of diseases should be cured at different levels of the hospital, which requires that the family doctors make an accurate diagnosis when patients first see a doctor. This kind of a tiered delivery system may achieve the goal of slight illness in the community, serious diseases in the hospital, and rehabilitation back to the community. The tiered delivery system meets the medical needs of patients with different types of disease and improves the quality and levels of medical services.

**Table 6:** The NMNL model empirical results (by disease types).

**Variables**	**Treatment outcome**		
	**Death**	**Transfer**	**No cure**
COH	0.4480^***^ (13.57)	1.8224^***^ (101.11)	−0.3668^***^ (−14.16)
Reimbursement ratio	−1.1874^***^ (−12.90)	−1.2968^***^ (−18.52)	6.3161^***^ (86.27)
Medical expenses	0.5749^***^ (28.24)	0.0738^***^ (4.45)	0.1094^***^ (7.35)
Gender	−0.45503^***^ (−17.91)	−0.0758^***^ (−4.41)	0.0714^***^ (5.32)
Age	0.0371^***^ (42.08)	0.0111^***^ (30.19)	−0.0031^***^ (−7.86)
Length of stay in hospital	−0.0001 (−0.12)	−0.1285^***^ (−41.15)	−0.0034^***^ (−3.42)
**Disease type**			
Critical disease	1.8656^***^ (19.62)	0.0730 (1.17)	0.8804^***^ (27.42)
Common disease	0.9320^***^ (10.19)	0.0442 (0.96)	−0.0111 (−0.40)
Constant term	−13.1579^***^ (−62.91)	−5.4037^***^ (−42.35)	−8.1251^***^ (−65.58)
Log likelihood	−178,250.54		
Prob>chi^2^	0.0000		
Pseudo *R*^2^	0.1111		

*** *Significant at 1%*.

### Robustness Check

After distinguishing COH and disease types, according to the empirical results (Table 7), the COH had a significant correlation with medical outcomes. Grade II Level A on death and transfer had a significant negative correlation with outcomes (*p* < 0.001), and no cure had significantly positive correlation (*p* < 0.001). The type of disease was associated with the medical outcomes. Taking the chronic disease as the reference group, the critical diseases on death and no cure were significantly positively (*p* < 0.001) associated with medical outcomes, which indicated that the severer the disease was, the higher the probability of death and transfer was. The Grade II Level A hospital played a limited role in curing the critical diseases. The Grade II Level B hospital had a significantly negative correlation with death and no cure (*p* < 0.001), and significantly a positive correlation with transfer (*p* < 0.001). This indicated that Grade II Level B hospital did not have the ability to admit and treat critical-diseased patients, which had a negative correlation with improving the medical outcome of critical-diseased patients.

**Table 7:** The NMNL model empirical results (by COH and disease types).

**Variables**	**Treatment outcome**								
	**Death**	**Transfer**	**No cure**	**Death**	**Transfer**	**No cure**	**Death**	**Transfer**	**No cure**
COH									
Grade II Level A hospitals	−0.3950^***^	−1.8733^***^	0.4337^***^						
	(−12.06)	(−106.58)	(16.73)						
Grade II Level B hospitals				−0.83304^***^	1.2154^***^	−0.3258^***^			
				(−14.39)	(68.98)	(−11.25)			
Grade II Level C hospitals							1.7362^***^	1.8946^***^	−0.3326^***^
							(44.81)	(68.4)	(−6.36)
Reimbursement ratio	−1.2352^***^	−1.1084^***^	6.2921^***^	−1.4661^***^	−1.1138^***^	6.3528^***^	−1.4778^***^	−1.2959^***^	6.4336^***^
	(−13.48)	(−16.47)	(86.06)	(−16.17)	(−16.75)	(86.6)	(−16.00)	(−19.73)	(88.19)
Medical expenses	0.5233^***^	0.0294*	0.1197^***^	0.4529^***^	−0.2578^***^	0.1512^***^	0.5635^***^	−0.2782^***^	0.1527^***^
	(29.22)	(1.85)	(8.09)	(25.05)	(−16.20)	(10.29)	(32.53)	(−17.64)	(10.41)
Gender	−0.4496^***^	−0.0728^***^	0.07211^***^	−0.4485^***^	−0.0455^***^	0.0720^***^	−0.4732^***^	−0.0444^***^	0.0733^***^
	(−17.72)	(−4.39)	(5.36)	(−17.67)	(−2.78)	(5.36)	(−18.54)	(−2.71)	(5.46)
Age	0.0368^***^	0.0082^***^	−0.0031^***^	0.0378^***^	0.0104^***^	−0.0036^***^	0.0341^***^	0.0111^***^	−0.0036^***^
	(43.99)	(21.25)	(−7.77)	(45.67)	(27.77)	(−9.07)	(41.1)	(30.08)	(−9.06)
Length of stay in hospital	0.0056^***^	−0.1241^***^	−0.0033^***^	0.0066^***^	−0.0942^***^	−0.0054^***^	0.0044^***^	−0.1023^***^	−0.0044^***^
	(8.9)	(−40.88)	(−3.37)	(9.32)	(−32.00)	(−5.27)	(9.48)	(−36.51)	(−4.37)
Disease type									
Critical disease	1.8814^***^	0.0479	0.8845^***^	1.9198^***^	0.0588	0.8728^***^	1.7944^***^	0.0506	0.8730^***^
	(19.79)	(0.79)	(27.25)	(20.20)	(0.98)	(27.2)	(18.83)	(0.84)	(27.2)
Common disease	0.9374^***^	0.0443	−0.0096	0.9145^***^	0.0549	−0.01658	0.8795^***^	0.0483	−0.0186
	(10.25)	(1.01)	(−0.34)	(10.46)	(1.27)	(−0.59)	(9.61)	(1.11)	(−0.66)
Constant term	−11.8746^***^	−1.4554^***^	−9.0015^***^	−11.4890^***^	−1.0766^***^	−8.8291^***^	−12.1370^***^	−0.6098^***^	−8.9325^***^
	(−65.90)	(−13.12)	(−79.06)	(−62.69)	(−9.54)	(−76.51)	(−68.30)	(−5.46)	(−78.26)
Log likelihood	−181,840.89	−185,328.86	−185,213.83
Prob>chi^2^	0.0000	0.0000	0.0000
Pseudo *R*^2^	0.1149	0.0980	0.0985

*** *Significant at 1%*.

Grade II Level C hospital on death and transfer was significantly positively associated with medical outcomes (*p* < 0.001), while no cure was significantly negatively associated (*p* < 0.001). Grade II Level C hospitals had a more significant negative correlation with improving the medical outcome of critical diseases. Grade II Level C hospitals had higher transfer rates for different disease types. However, compared with critically diseases, Grade II Level A hospitals and Grade II Level B hospitals played a role in improving the medical outcome of chronic diseases. In conclusion, hospitals of different levels should be reasonably selected for different types of diseases to achieve the optimal medical outcome. Meanwhile, after distinguishing COH and disease type, the empirical consequences of the MMNL model in Tables 4–6 showed that the regression results were relatively robust.

## Discussion

From the perspective of the COH framework, combing hospital characteristic factors, patient characteristic factors, and potential influencing factors, and using UEBMI data of Chengdu City from 2011 to 2015, our study empirically analyzed the COH correlation with medical outcomes by the nested multinomial logit modeling. This study had several important findings.

First, COH had a significant correlation with the medical outcomes, but the influence degree and direction of the COH on different outcomes were not consistent. COH had a significantly positive correlation with death and transfer and a significantly negative correlation with no cure, which may be related to the type and severity of the disease.

Second, reimbursement rate, health expenditures, gender, age, disease type, and others were the factors associated with the medical outcomes; length of stay influenced the medical outcome, but not all of them are uniformly significant, indicating that it was not that the longer the stay was, the better the outcome would be.

Third, after distinguishing COH, there were significant differences in the association with different levels of hospitals on the medical outcomes. Horizontally speaking, hospitals of the same grade had different correlations with different medical outcomes. There was competition between hospitals of the same grade. This result was similar to most of the previous literature which suggests that the hospital competition influences medical outcomes (47, 48). Different levels of hospitals had different correlations with the medical outcome. This shows that the medical outcome of big hospitals (high-level hospitals) is not necessarily better than that of small hospitals (low-level hospitals).

Finally, after distinguishing COH and disease types, the correlation with hospitals of different levels on the medical outcome of different disease types was significantly different. In terms of critical diseases, the higher-level hospital had a more significant correlation with improving the medical outcome, while the lower-level hospital had a significantly negative correlation with the medical outcome. However, for chronic diseases and common diseases, the high-level hospitals improving the medical outcome showed no significant correlation. Especially for chronic diseases, the correlation with low-level hospitals on improving the medical outcome was better than that of high-level hospitals. In conclusion, hospitals of different levels should be reasonably selected for different types of diseases to achieve the optimal medical outcome.

**Figure 3:**
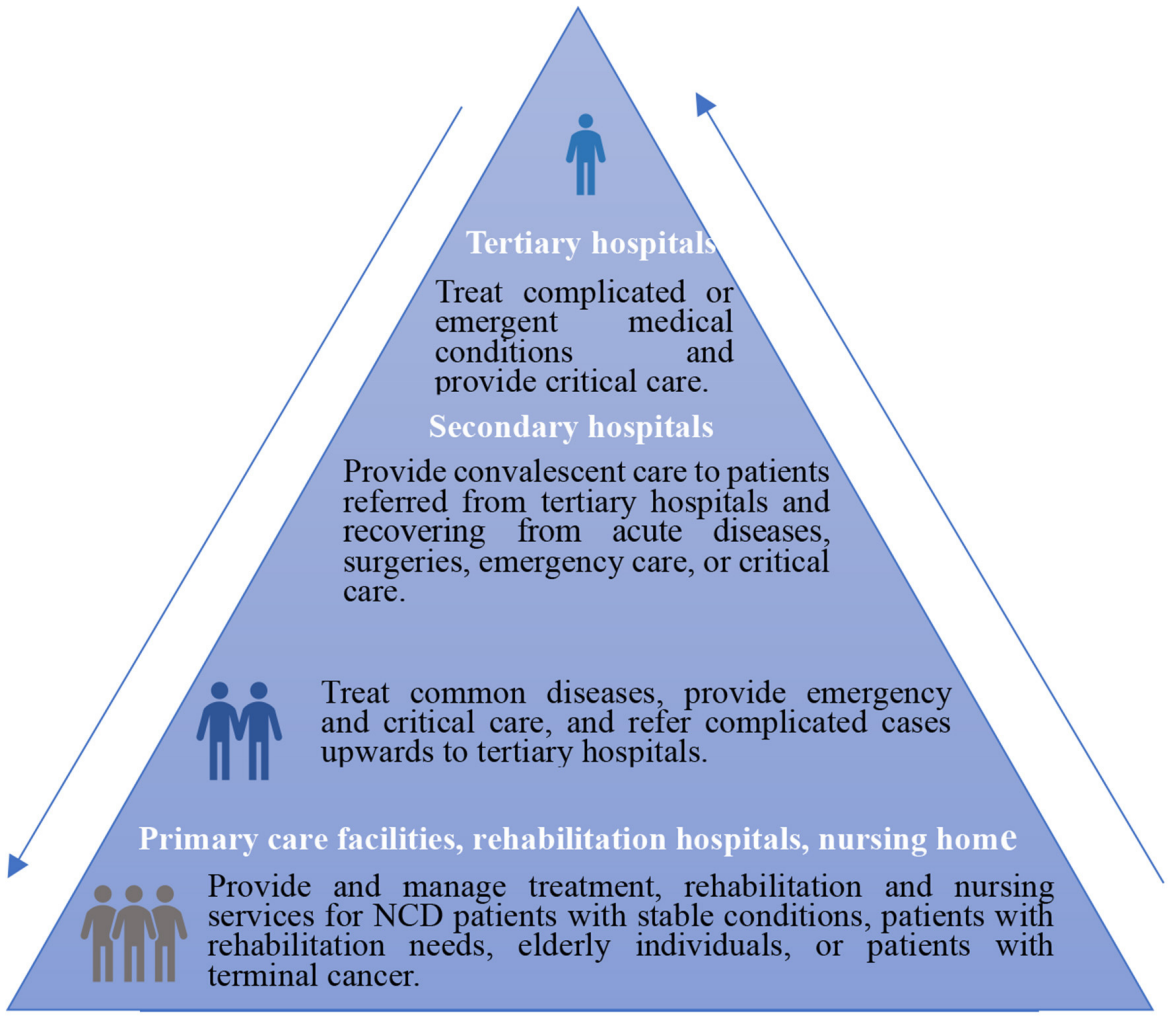
A tiered delivery system. According to the guidance of the general office of the state council on promoting a tiered delivery system. http://www.gov.cn/zhengce/content/2015-09/11/content_10158.html (accessed September 26, 2021).

Our study has some interesting findings that can potentially be used for policy recommendations. Therefore, according to Figure 3, the research about the effect of COH on medical outcomes proposes the following policy suggestions to promote the construction of a tiered delivery system: (a) the government should strengthen the publicity of a tiered delivery system and break the traditional idea and improve the service quality and service capacity of grassroots medical institutions to ensure the medical outcome. (b) The government should optimize the allocation structure of medical resources and promote the sinking of high-quality medical resources to the grass-roots level and provide more financial subsidies with grassroots-level medical institutions, encourage outstanding doctors to conduct multi-site practices and improve the ability and level of grassroots level medical institutions.

## Conclusion

In summary, this study measured the impact of different COH on medical outcomes using UEBMI data of Chengdu City from 2011 to 2015. Our study empirically analyzed the COH correlation with medical outcomes using the nested multinomial logit model. Our findings may contribute to the body of knowledge on the COH correlation with medical outcomes in China. Hospitals of different level have different influences on medical outcomes. In the process of attempting to improve the quality of medical service, the government addresses the medical insurance payment. Meanwhile, the government should also focus on promoting the construction of a tiered delivery system.

## Data Availability Statement

The raw data supporting the conclusions of this article will be made available by the authors, without undue reservation.

## Ethics Statement

Ethical review and approval was not required for the study on human participants in accordance with the local legislation and institutional requirements. Written informed consent from the [patients/ participants OR patients/participants legal guardian/next of kin] was not required to participate in this study in accordance with the national legislation and the institutional requirements.

## Author Contributions

LL and SZ carried out the study, analyzed the data, and drafted the manuscript. TD and LL was responsible for writing the literary and revising the language. LL provided the guidance for revising the manuscript. All authors read and approved the final manuscript.

## Funding

This project was funded by China Postdoctoral Science Foundation (No. 2022M713443) and the Research Funds of Renmin University of China (No. 2022K20166).

## Conflict of Interest

The authors declare that the research was conducted in the absence of any commercial or financial relationships that could be construed as a potential conflict of interest.

## Publisher's Note

All claims expressed in this article are solely those of the authors and do not necessarily represent those of their affiliated organizations, or those of the publisher, the editors and the reviewers. Any product that may be evaluated in this article, or claim that may be made by its manufacturer, is not guaranteed or endorsed by the publisher.
